# Animal Simples Approved for Modern Uses of Cure

**Published:** 1900-03

**Authors:** 


**Animal Simples Approved for Modern Uses of Cure.** By W. T. Fernie, M.D. Pp. xxiv., 565. Bristol: John Wright & Co. 1899.

This book is a companion to *Herbal Simples*, two editions of which we have noticed in these pages: like its forerunner, it displays much research and investigation. Dr. Fernie has industriously consulted many mediæval bestiaries and old books on medicine, and has compiled a work which possesses much interest, even if it is a little "snippety." A judicious revision of the text would have removed some of the paste-and-scissors quality and the occasional discrepancies. Medically the book is distinctly homœopathic, and some of the statements therefore will appear insufficiently established to those who are not of the author's persuasion, but for general instruction the work may be read by any one with profit.

